# Nanoscale-Precision Removal of Copper in Integrated Circuits Based on a Hybrid Process of Plasma Oxidation and Femtosecond Laser Ablation

**DOI:** 10.3390/mi12101188

**Published:** 2021-09-30

**Authors:** Shuai Wang, Yaoyu Wang, Shizhuo Zhang, Lingfeng Wang, Shuai Chen, Huai Zheng, Chen Zhang, Sheng Liu, Gary J. Cheng, Feng Liu

**Affiliations:** 1 Institute of Technological Sciences, Wuhan University, Wuhan 430072, China; shuai.wang@whu.edu.cn (S.W.); yaoyuwang@whu.edu.cn (Y.W.); danny-zhang@whu.edu.cn (S.Z.); wanglingfeng@whu.edu.cn (L.W.); shuaichen@whu.edu.cn (S.C.); c.zhang@whu.edu.cn (C.Z.); shengliu@whu.edu.cn (S.L.); 2 School of Power and Mechanical Engineering, Wuhan University, Wuhan 430072, China; huai_zheng@whu.edu.cn; 3 School of Industrial Engineering, Purdue University, West Lafayette, IN 47906, USA

**Keywords:** Cu removal, plasma oxidation, femtosecond laser ablation, roughness, nm

## Abstract

Copper (Cu) is the main interconnect conductor for integrated circuits (IC), and its processing quality is very important to device performance. Herein, a hybrid process of plasma oxidation and femtosecond laser (fs-laser) ablation was proposed for the nanoscale precision removal of Cu in integrated circuits. In this hybrid process, the surface layer of Cu was oxidized to the copper oxide by plasma oxidation, and then the fs-laser with a laser fluence lower than the Cu ablation threshold was used to remove the copper oxide without damaging the underlying Cu. Theoretically, the surface temperature evolutions of Cu and copper oxide under the femtosecond laser were studied by the two-temperature model, and it was revealed that the ablation threshold of copper oxide is much lower than that of Cu. The experimental results showed that the ablation threshold of copper oxide is lower than that of Cu, which is consistent with the theoretical analysis. Using the hybrid process, a surface roughness of 3 nm and a removal accuracy of 4 nm were obtained in the process of Cu film processing, which were better than those obtained by fs-laser ablation. This demonstrated that the hybrid process has good application potential in the field of copper micromachining.

## 1. Introduction

Copper (Cu) is widely used in the fields of optics and micro-nano devices due to its low resistivity and superior resistance to electromigration, which can meet the requirements of high speed and low power consumption of the chip [1,2,3,4,5]. With the development of the integrated circuits (IC), nanometer surface roughness is required to adapt to the reduction in device size and the focal depth of lithography equipment [6,7,8,9,10]. Cu, as a common interconnection conductor in an IC, is required to have nanometer-level surface quality in IC applications. In the IC process, Cu film is mainly deposited by physical vapor deposition (PVD) and chemical vapor deposition (CVD) [11,12], and the removal of Cu is realized by chemical mechanical polishing (CMP) [13,14,15]. However, due to a soft metal material, Cu is prone to form surface defects such as scratches, dishing, and erosion during CMP [16]. Besides, the chemical reaction between the slurry and Cu can also lead to excessive surface corrosion problems such as disk pits or erosion pits [17], and CMP slurry can cause environmental pollution problems. Dry etching is also commonly used to etch Cu film with halogen gases such as CCl_4_, SiCl_4_, Cl_2_, and HBr [18,19]. Nevertheless, dry etching requires expensive vacuum equipment, and the emission of reaction gas also needs to be properly treated. Therefore, it is necessary to develop a low-cost, low-defect, low-pollution, and high-precision Cu removal process for ICs.

Femtosecond laser (fs-laser) ablation has been demonstrated as an effective means for the micromachining and surface modification of solid materials because of its high machining accuracy, low cost, small heat-affected zone, and controllable and noncontact characteristics, which has been widely used in the field of Cu micromachining [20,21,22]. For example, many studies have been made on welding [23], drilling [24], cutting [25], and surface modification [26,27,28] by the fs-laser. However, surface defects such as pits, cracks, and molten particles are easy to occur during the fs-laser ablation [29], which makes it difficult to achieve nanometer-level processing quality. Obviously, laser ablation cannot meet the process requirements of Cu nano-precision removal in ICs.

In this study, to obtain nanoscale Cu removal precision, a hybrid process of plasma oxidation and fs-laser ablation was proposed. In this process, using the difference in ablation thresholds between Cu and copper oxide, the fs-laser removes copper oxide without ablating the underlying Cu, to achieve self-limiting Cu removal. First, combining the two-temperature model (TTM) with the finite element analysis (FEA), the surface temperature evolutions of Cu and Cu_2_O under the fs-laser irradiation were investigated, and the difference in the ablation thresholds of Cu and Cu_2_O was revealed. Then, experimental studies on the removal of the Cu layer on the silicon substrate by the fs-laser ablation and the hybrid process were carried out. The experimental results showed that the hybrid process with low laser energy could effectively remove the Cu layer and achieve nanoscale processing accuracy. The surface of the Cu film ablated by the fs-laser had obvious ablation traces, which leads to the increase in surface roughness and the decline in surface quality. It could be seen that the hybrid process can better meet the requirements of Cu processing in ICs than the fs-laser ablation can.

## 2. Experiment

### 2.1. Materials

The silicon (Si) substrate (thickness of 430 μm, surface roughness < 0.5 nm) was ultrasonically cleaned in acetone, ethanol, and deionized water (Sinopharm Chemical Reagent Co., Ltd., Shanghai, China) for 15 min to remove surface impurities. Then, a copper film with a thickness of 1.5 μm was deposited on the silicon substrate by the thermal evaporation process.

### 2.2. Laser Configuration

**Figure 1:**
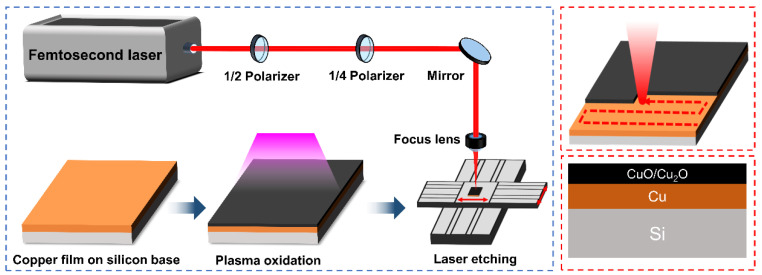
Schematic diagram of experimental program.

The Cu removal hybrid process is schematically illustrated in Figure 1. The surface of the Cu film was oxidized to copper oxide by an oxygen plasma generator (Zepto-BRS-W6, Diener electronic GmbH & Co. KG, Ebhausen, Germany), and the thickness of the oxide layer was controlled by adjusting the treatment time. A 1035 nm fs-laser (Monaco 1035-80-60-C, Coherent, CA, USA) with a pulse duration of 270 fs and repetition rate of 15 kHz was used as a light source to irradiate the sample in the air. The sample was shifted by a two-dimensional (2D) (X, Y) motion platform to realize the laser-selective region scanning according to the pre-designed scanning paths. The overlap ratio of the laser spot was set to 60% in the X and Y directions of the irradiation area.

### 2.3. Characterization

The surface morphology and element content of the Cu films were observed and analyzed by a scanning electron microscope (SEM) (Zeiss Sigma, Carl Zeiss AG Co. Ltd., Oberkochen, Germany). The 3D morphology and roughness of the Cu surface were characterized by an optical profiler (NewView 9000, Zygo, Middlefield, CT, USA). The chemical composition of the sample was measured with confocal Raman microspectroscopy (RTS2, Zolix Instruments Co., Ltd., Beijing, China).

## 3. Modeling Approach and Numerical Simulation

### 3.1. Model

The ablation mechanism of the fs-laser is different from that of traditional long-pulse lasers and there is a strong nonlinear effect in the interaction between the fs-laser and materials. The interaction time between the fs-laser and metal is less than the coupling time between the electron and lattice subsystems, so the extremely unbalanced state in the fs-laser ablation can be analyzed using a TTM that describes the interaction among the laser, electron, and lattice systems [21,25].

(1) $$\begin{array}{c} C_{e}\frac{\partial T_{e}}{\partial t}=\nabla \left(k_{e}\nabla T_{e}\right)-G\left(T_{e}-T_{l}\right)+S_{\left(r,z,t\right)} \end{array}$$

(2) $$C_{l}\frac{\partial T_{l}}{\partial t}=\nabla \left(k_{l}\nabla T_{l}\right)+G\left(T_{e}-T_{l}\right)$$

where C is the heat capacity, k is the thermal conductivity, and e and l denote the electron and lattice, respectively. G is the electron–phonon coupling factor, t is the time, and z is the direction perpendicular to the target surface. In this work, the lattice heat capacity is considered to be a constant $C_{l}=\rho c_{P}$, and the electron heat capacity is taken as being proportional to electron temperature $C_{e}=B_{e}T_{e}$ [30].

(3) $$\begin{array}{c} B_{e}=\frac{\pi^{2}n_{e}k_{B}}{2T_{F}} \end{array}$$

where ρ is the density, $c_{P}$ is the specific heat capacity, $n_{e}$ is the electron number density, $k_{B}$ is Boltzmann’s constant, and $T_{F}$ is the Fermi temperature. The electron–phonon coupling coefficient represents the heat energy exchange efficiency between electrons and phonons in unit volume and can be estimated by the following equation [30]:

(4) $$\begin{array}{c} G=\frac{9}{16}\frac{n_{e}k_{B}^{2}T_{D}^{2}\nu_{F}}{\Lambda \left(T_{l}\right)T_{l}\mu_{F}} \end{array}$$

where $T_{D}$ is the Debye temperature, $\nu_{F}$ is the Fermi velocity, Λ is the average electron free path, and $\mu_{F}$ is the Fermi energy. The thermal diffusion of the electron subsystem is much faster than that of the lattice, and the electron thermal conductivity is related to both the electron and lattice temperatures under nonequilibrium [31].

(5) $$\begin{array}{c} k_{e}=\chi \frac{{\left(\emptyset_{e}^{2}+0.16\right)}^{\frac{5}{4}}\left(\emptyset_{e}^{2}+0.44\right)\emptyset_{e}}{{\left(\emptyset_{e}^{2}+0.092\right)}^{\frac{1}{2}}\left(\emptyset_{e}^{2}+\beta \emptyset_{l}\right)} \end{array}$$

where $\emptyset_{e}=\frac{T_{e}}{T_{F}}$ and $\emptyset_{l}=\frac{T_{l}}{T_{F}}$. χ and β are material constants. In the 2D axisymmetric TTM established in this work, the laser source S can be expressed for a single pulse as:

(6) $$\begin{array}{c} S_{\left(r,z,t\right)}=I_{\left(r,z,t\right)}\alpha \left(1-R\right) \end{array}$$

where $I_{\left(r,z,t\right)}$ is the power density function of the laser, R is the reflectivity of the material surface to the laser, and α is the absorption coefficient of the material (the reciprocal of the optical penetration depth δ of the laser). The attenuation of material to the laser obeys the Lambert–Beer law. The output of the Gaussian heat source can be defined as follows [25]:

(7) $$\begin{array}{c} I_{\left(r,z,t\right)}=-0.94\frac{F}{t_{P}}\exp\left(\frac{-2r^{2}}{r_{0}{}^{2}}\right)\exp\left[-\int_{0}^{z}\frac{1}{\delta }d_{z}-4ln2{\left(\frac{t-t_{P}}{t_{P}}\right)}^{2}\right] \end{array}$$

where F is the laser energy density and $t_{P}$ is the pulse duration. $r_{0}$ is the radius of the laser beam and r is the distance of a point measured from the laser beam center.

### 3.2. Simulation

Due to the nonlinearity of the coupled heat conduction equations, the finite element method was adopted to solve the above equations in this study. The mesh around the laser beam was locally refined and there were a total of 3450 mesh cells in the whole domain. In each step, the iterative method was adopted to solve the electron and lattice energy equations, and the material parameters involved in the equations were updated. The deformed mesh was used to simulate the ablation process of the material after laser irradiation. The ablation rate of the solid boundary can be expressed as: $v_{a}=\frac{h_{a}\left(T_{l}-T_{a}\right)}{\rho H_{s}}$. $H_{s}$ is the heat of evaporation. When the lattice temperature $T_{l}$ is higher than the evaporation temperature $T_{a}$, mesh displacement occurs and the material is considered to be removed.

## 4. Results and Discussion

### 4.1. Simulation Results

The main content of copper oxide generated in low-power oxygen plasma is Cu_2_O, which is related to the low activation energy required for the formation of Cu_2_O [32]. Therefore, the theoretical models of the fs-laser interaction with Cu and Cu_2_O were established to simulate the ablation of samples by a single-pulse fs-laser with different laser fluences. The physical properties [33,34,35,36,37] of Cu and Cu_2_O are listed in Table 1.

**Figure 2:**
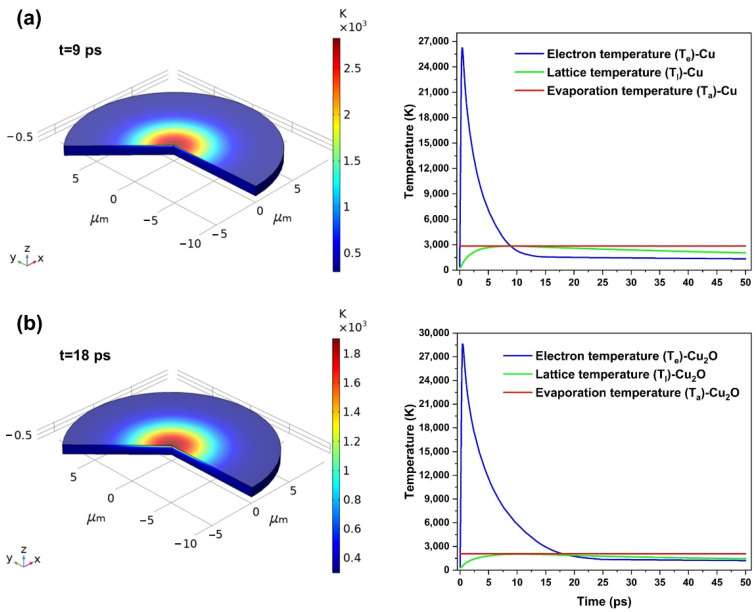
The distribution of lattice temperature and the evolution of electrons and lattice temperature as a function of time during the irradiation in the single pulse: (**a**) Cu and (**b**) Cu_2_O.

The ablation thresholds of Cu and Cu_2_O are calculated as 0.89 J/cm^2^ and 0.37 J/cm^2^, respectively, by the simulations. Figure 2 shows the lattice temperature distribution of Cu irradiated by the fs-laser in the single pulse with 0.89 J/cm^2^ and Cu_2_O irradiated by the fs-laser in the single pulse with 0.37 J/cm^2^ laser fluences, as well as the evolution of electron and lattice temperature as a function of time at the spot center. The electron temperature reaches a very high value immediately after absorbing the photon energy, and the vibration of the lattice is slower than the electron transition, which requires the absorption or release of phonons to reach a relative thermal balance with the electron in the picosecond scale. Surface ablation occurs when the lattice temperature $T_{l}$ reaches the evaporation temperature $T_{a}$ of the corresponding material. According to the simulation results of lattice temperature under different laser fluences, single-shot ablation thresholds of Cu and Cu_2_O around 0.89 J/cm^2^ and 0.37 J/cm^2^ are obtained, respectively. Therefore, it is expected that the oxide layer (Cu_2_O) on the Cu substrate can be effectively removed without damage to the Cu substrate under the appropriate laser fluence.

### 4.2. Laser Ablation of Cu Films

**Figure 3:**
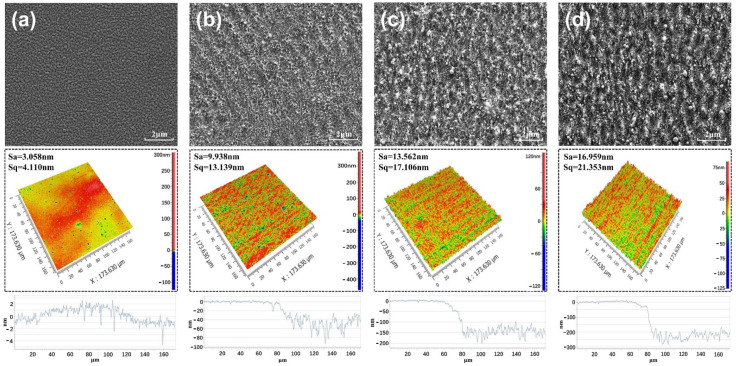
Cu films’ surfaces irradiated by the femtosecond laser (fs-laser) with different laser fluences: (**a**) untreated, (**b**) 0.53 J/cm^2^, (**c**) 0.61 J/cm^2^, and (**d**) 0.7 J/cm^2^.

The influence of laser energy density on the surface morphology of ablated Cu film was studied. The surface morphology of the fs-laser-ablated Cu film is shown in Figure 3. It can be seen from Figure 3a that the Cu film without the fs-laser treatment has a uniform surface with a surface roughness of about 3 nm. As shown in Figure 3b, when the laser fluence is 0.53 J/cm^2^, the uniformly periodic rippled structures, and many small-sized nanoparticles can be observed on the Cu surface, indicating that Cu is initially ablated by the fs-laser. Under the single-pulse irradiation, the threshold fluence of the copper film is consistent with the simulation results (0.89 J/cm^2^). Nevertheless, it decreases to 0.53 J/cm^2^ as more pulses and laser energy are deposited per unit area, which indicates an accumulative behavior. A major factor in the formation mechanisms of the periodic surface structures observed in Figure 3b is considered to be the interference caused by an incident laser beam with the laser-excited surface plasmon wave [21]. In addition, its period has obvious wavelength-dependent characteristics (slightly smaller than the incident laser wavelength), which is in good agreement with the experimental results [38]. With the increase in laser fluence, the nonuniformity of the ablated Cu surface becomes more serious due to the enhancement of plasma shock and thermal effect. In Figure 3c,d, the irregular laser deposition products and many micron-sized molten particles formed by ablating can be seen on the surface of the Cu film, and the obvious vaporized and re-solidified traces of materials occur in the entire irradiated area. Meanwhile, the surface roughness and ablation depth of the Cu film increase almost linearly with the laser fluence. This is because within the limit of plastic deformation of the material, the degree of material damage becomes more serious with the increase in laser energy. When the laser fluence increases from 0.53 J/cm^2^ to 0.7 J/cm^2^, the surface roughness and average removal thickness of the Cu film increase from 9.938 nm to 16.959 nm, and from 60 nm to 250 nm, respectively. It can be seen that the higher the energy density of the Gauss laser beam, the more serious the ablation of the Cu film surface.

### 4.3. Oxidation-Laser Ablation

#### 4.3.1. Surface Morphology and Composition

**Figure 4:**
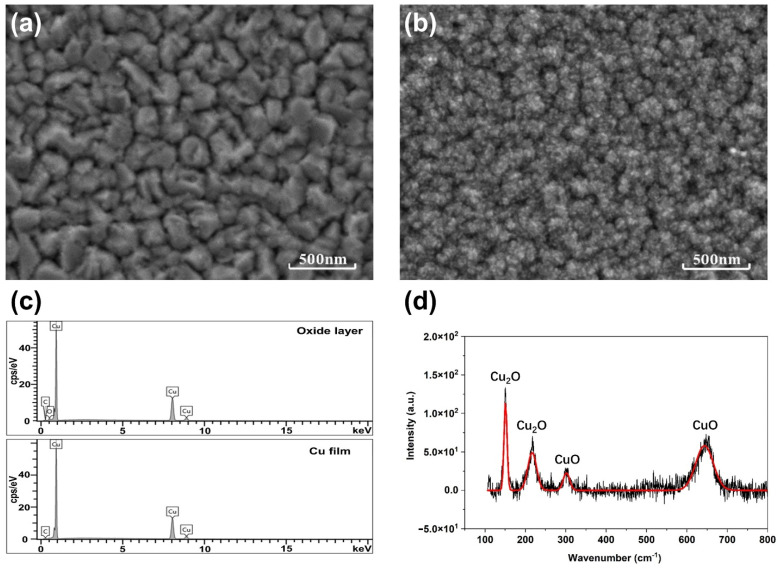
Scanning electron microscope (SEM) images of surface morphology of (**a**) the Cu film and (**b**) the copper oxide layer. (**c**) EDS spectra of the Cu film and the copper oxide layer. (**d**) Raman spectrum of the copper oxide layer.

The Cu film on the Si substrate was oxidized with oxygen plasma. The effect of plasma oxidation on the surface chemical composition and morphology of the Cu film was investigated, as shown in Figure 4. In Figure 4a, the Cu film deposited on the Si substrate grows layer by layer via island nucleation. After the plasma oxidation treatment with a radio frequency (RF) power of 90 W, a layer of fine oxide particles appears on the surface of the Cu film, as shown in Figure 4b. This indicates that the surface copper of the Cu film is oxidized to copper oxide. The energy dispersive spectroscopy (EDS) spectra in Figure 4c also show that the oxygen content on the surface of the sample appears correspondingly after the oxygen plasma treatment. A confocal Raman system was used to analyze the composition of the oxide layer. The characteristic peaks corresponding to vibrational Raman modes of Cu_2_O and CuO are observed in Figure 4d, indicating that the oxide is composed of Cu_2_O and CuO.

**Figure 5:**
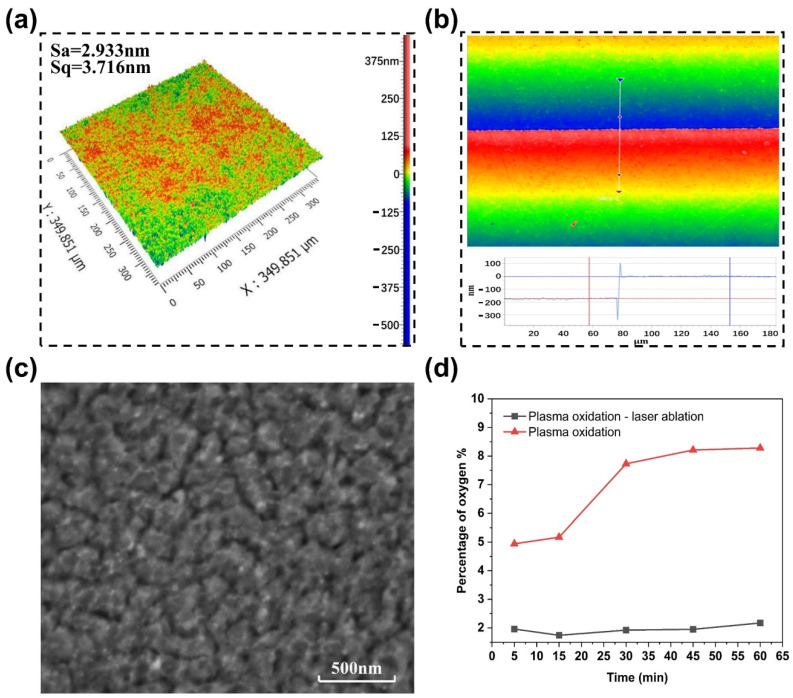
Surface morphology of the Cu film obtained by the hybrid removal process. (**a**) three-dimensional (3D) morphology, (**b**) the boundary between untreated and laser-treated areas, and (**c**) SEM image. (**d**) Evolution of the surface oxygen content.

The fluence (0.39 J/cm^2^), whose value is lower than the ablation threshold of Cu, was used to irradiate the copper oxide layer. The fluence value, in this case, could be beneficial to effectively remove the copper oxide layer without damaging the underlying copper and realize a self-limiting strategy. As shown in Figure 5a, the Cu film with a roughness of 2.933 nm is obtained after the oxide layer is removed by the fs-laser ablation, which is the same as the original Cu film in Figure 3a. It indicates that the smooth surface morphology of Cu film is not damaged when the oxide layer is removed by a low laser fluence, which can meet the requirements of integrated circuits for copper surface roughness. While obtaining the high surface quality of the Cu film, the oxide layer with a thickness of about 172 nm was removed by the fs-laser (Figure 5b), indicating the high efficiency of the hybrid process. Figure 5c shows that the treated Cu film has similar structural characteristics to that of the original Cu film, and no molten particles and ablative traces appear on the sample surface. The oxygen contents of Cu film before and after the fs-laser ablation were analyzed, as shown in Figure 5d. In the figure, as the plasma oxidation time increases, the oxygen content in the Cu film gradually increases to a saturation value of 8%. After the oxide layer is removed by the fs-laser, the oxygen content in the Cu film decreases rapidly to about 2%. The existence of a weak oxygen signal is due to the natural oxidation caused by the thermal effect when the oxide is removed by the fs-laser. The natural oxide layer is usually very thin (several nanometers) and does not effect on the conductivity of the Cu film. In short, it can be seen from the above experimental results that using the difference in fs-laser ablation thresholds between copper oxide and copper, the hybrid process removes Cu in a two-step method, thereby obtaining a surface with nano-level roughness. Compared with the direct ablation of copper using the fs-laser (Figure 3), the hybrid process achieves better copper processing quality.

#### 4.3.2. Removal Accuracy

In the hybrid process, the ablation depth of Cu is determined by the thickness of the copper oxide layer.

Because the nanoscale controllable growth thickness of the oxide layer can be achieved by adjusting the power and the irradiation time of the oxygen plasma, the nano-precision removal of Cu can be realized by the hybrid process.

**Figure 6:**
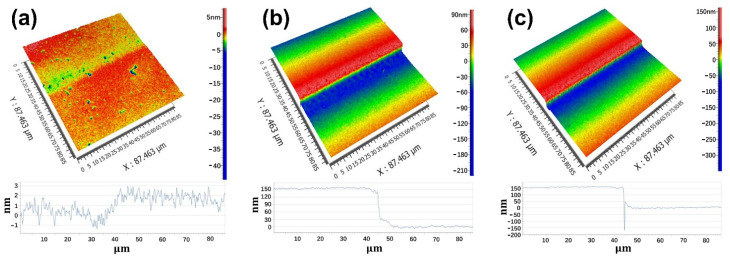
Laser ablation depth under different oxidation durations: (**a**) 5 min, (**b**) 30 min, and (**c**) 60 min.

The thickness of the oxide layer on the surface of the copper film is controlled by adjusting the oxidation time of Cu in oxygen plasma, and the oxide layer is removed by the fs-laser with the same laser fluence (0.39 J/cm^2^). When the oxidation time is 5 min, the laser ablation depth of copper is only about 4 nm, as shown in Figure 6a. With the increase in oxidation time, the thickness of the removed copper layer increases gradually. When the oxidation time is 30 min and 60 min, the copper film is correspondingly thinned by 156.7 nm in Figure 6b and 163.6 nm in Figure 6c, respectively, after the oxide layer is completely removed. Moreover, the surface roughness of the copper film remains unchanged after femtosecond laser ablation. In addition, it can be seen from the figure that the oxidation time increases to a certain extent, and the increase in the thickness of the oxide layer becomes very slow. This is due to the fact that when the oxide layer grows to a certain thickness, the oxide layer will prevent oxygen from entering the interface between the copper and the oxide layer for further oxidation. The changing trend is also verified by the test results of the oxygen content in the copper film in Figure 5d.

**Figure 7:**
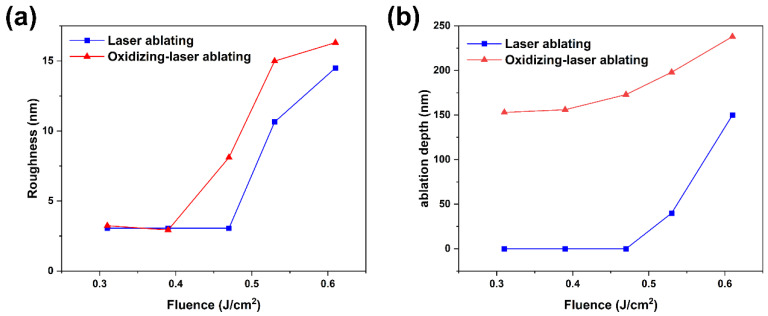
(**a**) Roughness and (**b**) ablation depth of the Cu films obtained by laser ablation and hybrid process under different laser fluences.

Figure 7 shows the surface roughness and ablation depth of untreated and plasma-oxidized (90 W, 30 min) Cu films as a function of laser fluence.

When the incident fluence is less than 0.39 J/cm^2^, the Cu film treated by the hybrid process obtains a surface roughness of 3 nm with a removal depth of 156.7 nm, and the Cu film treated by the fs-laser ablation does not change, because the fluence is lower than the ablation threshold of Cu. When the laser fluence increases to 0.47 J/cm^2^, the surface roughness and the ablation depth of the Cu film treated by the hybrid process increase further, indicating that the Cu layer under the oxide layer is also ablated even if the fluence is lower than the ablation threshold of Cu film. It is related to the low thermal conductivity and high light absorption of the oxide layer [36], resulting in more absorption for laser energy. Compared with pure copper, the oxide has a lower thermal conductivity and porous structure. The existence of pores leads to air infiltration, which further reduces the surface heat transfer performance. Therefore, it is easy to cause local overheating to form hotspots. The thermal effect generated by high fluence cannot be alleviated, and the existence of these hotspots in processing leads to severe damage to the underlying material, which increases the surface roughness and ablation depth. When the laser fluence is higher than the copper ablation threshold, the Cu film treated by laser ablation begins to change, and the surface roughness reaches more than 10 nm and the Cu layer with hundreds of nanometers is removed. Therefore, the hybrid removal process of Cu film proposed in this work can achieve the desired effect under low laser fluence.

## 5. Conclusions

In this study, a hybrid process of plasma oxidation and fs-laser ablation was proposed to obtain nanoscale surface roughness in the process of Cu film micromachining. Combining the two-temperature model with the finite element analysis (FEA), it was confirmed theoretically that the ablation threshold of copper oxide is much lower than that of Cu, which was also verified by experimental study. Based on the difference in the fs-laser ablation thresholds between Cu and copper oxides, the upper copper oxides can be selectively removed without damaging the underlying Cu under irradiation of the fs-laser with low laser fluence. In the experiment of removing the Cu layer on the silicon substrate by the fs-laser ablation, the surface roughness of the Cu film ablated by the fs-laser increased to more than 10 nm, indicating the decline in the Cu surface quality. However, using the hybrid process, a surface roughness of 3 nm and a removal accuracy of 4 nm were obtained in the process of Cu film processing, which was better than those obtained by laser ablation. The Cu removal hybrid process with high smoothness and accuracy provides a new idea for Cu micromachining in the electronic industry and precision devices.

## Figures and Tables

**Table 1:** Physical properties of Cu and Cu_2_O.

Species	Density (g/cm^3^)	Reflectivity	**Absorption Coefficient** $\left({\mu m}^{-1}\right)$	Thermal Conductivity (W/(m·K))	Evaporation Temperature (K)
Cu	8.96	0.9	70.8	377	2835
Cu_2_O	6.09	0.21	1.1	7	2073
